# Identification and Analysis of MicroRNAs Associated with Wing Polyphenism in the Brown Planthopper, *Nilaparvata lugens*

**DOI:** 10.3390/ijms21249754

**Published:** 2020-12-21

**Authors:** Le Xu, Jiao Zhang, Anran Zhan, Yaqin Wang, Xingzhou Ma, Wencai Jie, Zhenghong Cao, Mohamed A. A. Omar, Kang He, Fei Li

**Affiliations:** 1 State Key Laboratory of Rice Biology & Ministry of Agricultural and Rural Affairs Key Laboratory of Molecular Biology of Crop Pathogens and Insect Pests, Institute of Insect Sciences, Zhejiang University, Hangzhou 310058, China; xule1208@zju.edu.cn (L.X.); zhananran@zju.edu.cn (A.Z.); zxmlmq@163.com (X.M.); 3130100574@zju.edu.cn (Z.C.); dr.abdelwanees@alexu.edu.eg (M.A.A.O.); lifei18@zju.edu.cn (F.L.); 2 College of Plant Protection, Nanjing Agricultural University, Nanjing 210095, China; 15064210267@139.com (J.Z.); jiewencai@gmail.com (W.J.); 3 State Key Laboratory of Rice Biology, Institute of Biotechnology, Zhejiang University, Hangzhou 310058, China; wangyq0219@126.com; 4 Department of Plant Protection, Faculty of Agriculture (Saba Basha), Alexandria University, Alexandria 21531, Egypt

**Keywords:** microRNA, wing polyphenism, insulin, hormone

## Abstract

Many insects are capable of developing two types of wings (i.e., wing polyphenism) to adapt to various environments. Though the roles of microRNAs (miRNAs) in regulating animal growth and development have been well studied, their potential roles in modulating wing polyphenism remain largely elusive. To identify wing polyphenism-related miRNAs, we isolated small RNAs from 1st to 5th instar nymphs of long-wing (LW) and short-wing (SW) strains of the brown planthopper (BPH), *Nilaparvata lugens*. Small RNA libraries were then constructed and sequenced, yielding 158 conserved and 96 novel miRNAs. Among these, 122 miRNAs were differentially expressed between the two BPH strains. Specifically, 47, 2, 27 and 41 miRNAs were more highly expressed in the 1st, 3rd, 4th and 5th instars, respectively, of the LW strain compared with the SW strain. In contrast, 47, 3, 29 and 25 miRNAs were more highly expressed in the 1st, 3rd, 4th and 5th instars, respectively, of the SW strain compared with the LW strain. Next, we predicted the targets of these miRNAs and carried out Gene Ontology and Kyoto Encyclopedia of Genes and Genomes pathway analysis. We found that a number of pathways might be involved in wing form determination, such as the insulin, MAPK, mTOR, FoxO and thyroid hormone signaling pathways and the thyroid hormone synthesis pathway. Thirty and 45 differentially expressed miRNAs targeted genes in the insulin signaling and insect hormone biosynthesis pathways, respectively, which are related to wing dimorphism. Among these miRNAs, *Nlu-miR-14-3p*, *Nlu-miR-9a-5p* and *Nlu-miR-315-5p*, were confirmed to interact with *insulin receptor*s (*NlInR*s) in dual luciferase reporter assays. These discoveries are helpful for understanding the miRNA-mediated regulatory mechanism of wing polyphenism in BPHs and shed new light on how insects respond to environmental cues through developmental plasticity.

## 1. Introduction

Polyphenism, which occurs when organisms share the same genotype but exhibit two or more distinct phenotypes, is crucial for organisms to successfully deal with heterogeneous environments. This ecological phenomenon may be determined by multiple environmental conditions, including temperature, diet and population density [1]. For instance, the southern African butterfly, *Bicyclus anynana*, develops into a dry season form when exposed to a low temperature during the larval stage [2] and female honeybees, depending on the composition of nutrients they are fed as larvae, develop into two different castes, exhibiting a large degree of variation in morphology, physiology and social function [3,4]. Depending on the local population density, locusts transform between two phases, a low-density “solitarious” phase and a high-density “gregarious” phase [5,6]. Wing polyphenism is well known in insects, with distinct wing phenotypes conferring differential dispersal abilities [7,8,9]. Long-wing (LW) individuals with fully developed wings and flight muscles are capable of dispersing to new habitats, while short-wing (SW) or wingless individuals allocate resources to reproduction rather than to flight [1,10]. 

The brown planthopper (BPH), *Nilaparvata lugens* (stål) (Hemiptera: Delphacidae) shows obvious wing polyphenism in response to environmental cues [10]. This capability enables the BPH to migrate over long distances and because of this, it has become a notorious rice pest in Asia. The insulin/insulin-like growth factor (IGF) signaling (IIS) pathway participates in the determination of wing morphs in BPHs [11]. Specifically, two insulin receptors, InR1 and InR2, play opposing roles in controlling the development of wings by regulating the activity of the master transcription factor, FoxO [11,12]. Wounding and host quality also affect the development of wing morphs via regulation of the IIS pathway [13,14]. With high-quality host plants, nymphs will develop into the SW form through the induction of *Ultrabithorax* (*NlUbx*) expression [15]. Moreover, insect hormones, including juvenile hormones (JHs) and ecdysone, also act as important factors in wing-morph determination [10]. High titers of JH in the penultimate nymphal instar induce BPH nymphs to develop into SW adults [16], while silencing *JH epoxide hydrolase* (*Nljheh*), a metabolic enzyme catalyzing JH degradation, increases the ratio of SW individuals in the population [16]. As a key endocrine signal, ecdysone modulates insect molting and metamorphosis but also participates in wing morph determination in the aphid *Acyrthosiphon pisum* [17,18]. Multiple signaling pathways are involved in regulating wing morph determination and dozens of genes show differences in stage-dependent expression between LW and SW BPHs [19]. However, how these genes work together in the associated pathways to modulate polyphenism is still unknown. 

MicroRNAs (miRNAs) are small non-coding RNA molecules (~22 nt) that function in posttranscriptional repression of gene expression in diverse eukaryotic lineages [20]. Numerous studies have shown that miRNAs play roles in a complex regulatory system for biological processes [21,22,23,24,25,26]. In insects, miRNAs are involved in the regulation of growth, metamorphosis and reproduction [27,28,29,30,31,32]. For instance, multiple miRNAs jointly regulate the metamorphosis development via the biosynthesis of 20E in *Chilo suppressalis* [27]. Additionally, miRNAs also function in the phenotypic plasticity. In the locust, *miR-133* inhibition resulted in the behavioral shift from the solitary phase to the gregarious phase [30]. Overexpression of *miR-9b* facilitated the development of wing and reduced the proportion of winged offspring in aphids [31]. Recently, *Nlu-miR-34* was found to mediate cross talk between the IIS pathway and two hormones, JH and 20-Hydroxyecdysone (20E), by targeting *InR1* and forming a positive feedback loop [32]. Given that multiple factors regulate wing polyphenism in BPH, we hypothesize that miRNAs can indirectly control wing morph plasticity by regulating these key determinants, including factors involved in signal transduction pathways and hormone biosynthesis. Here, we constructed and sequenced small RNA libraries of nymph BPHs at the 1st to 5th instar stages for both the LW and SW strains. Differentially expressed miRNAs between the two strains were identified and the targets of these miRNAs were predicted. Gene Ontology (GO) and Kyoto Encyclopedia of Genes and Genomes (KEGG) pathway enrichment analyses were performed to annotate the functions and pathways involved in the regulation of wing polyphenism. Some miRNAs were also selected to validate the interactions with targets. Our results reveal a complicated miRNA network that potentially determines the stage-dependent modulation of wing morph plasticity in BPH. This network increases our understanding of how miRNA regulates wing polyphenism and should be helpful for developing novel methods for controlling BPH in the future.

## 2. Results

### 2.1. Identification of miRNAs in BPH

**Figure 1:**
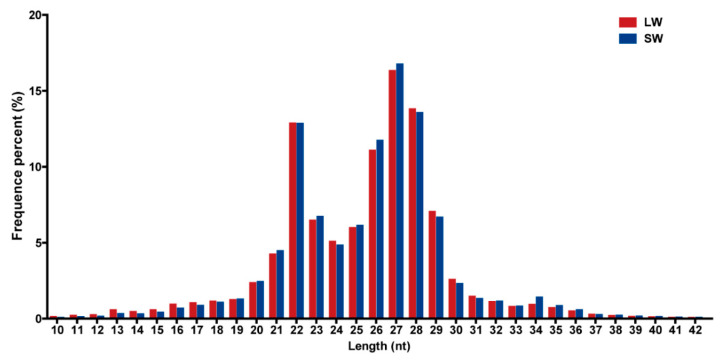
Distribution of total small RNA read lengths in long-wing (LW) and short-wing (SW) brown planthopper (BPH) strains.

To identify miRNAs associated with the development of wing morph plasticity, we first pooled 20 small RNA libraries constructed with RNA isolated from 1st to 5th instar nymphs of LW and SW BPH strains with two replicates per sample. After removing the low-quality sequences, 148,129,963 and 160,959,695 clean reads were obtained for the LW and SW strains, respectively (Table 1). The distributions of total small RNA read lengths were similar with a distinct bimodal peak (one peak at 21–23 nt and another at 26–29 nt) in both strains (Figure 1). To identify and annotate the full repertoire of miRNAs in BPH, we used two different algorithms, miRDeep2 [33,34] and MapMi [35], to predict conserved and novel miRNAs. In total, we identified 254 miRNAs (158 conserved and 96 novel miRNAs) in the small RNA libraries, which were combined before miRNA prediction (Supplementary Table S1). The conserved miRNAs belong to 60 known miRNA families, while the novel miRNAs belong to 42 unknown miRNA families. The number of miRNAs identified here is consistent with the number identified in BPH in previous studies [36,37] and these miRNAs were used for further analysis.

### 2.2. Identification of Wing Morph-Related miRNAs in BPH

**Figure 2:**
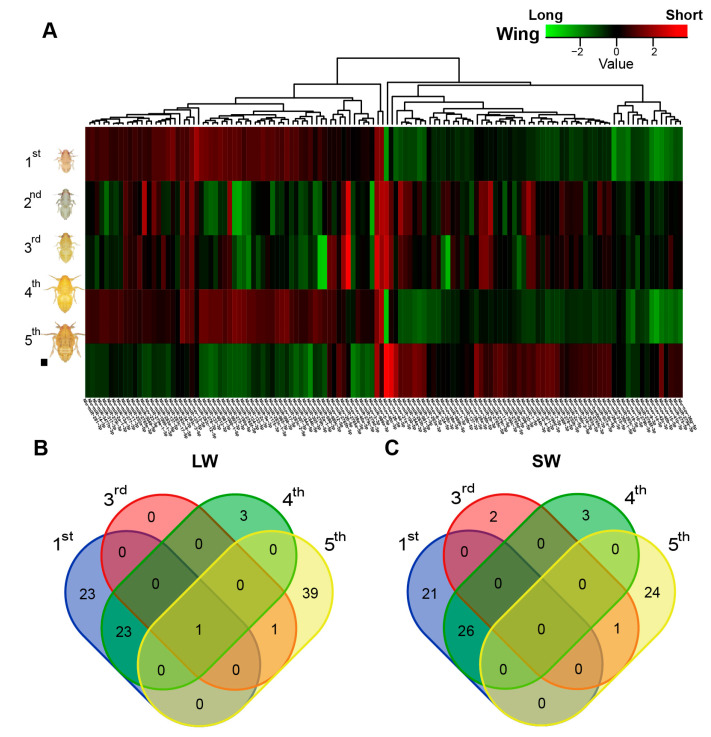
Differentially expressed miRNAs between LW and SW strains. (**A**) Heatmap of differentially expressed miRNAs between LW and SW strains. Green indicates miRNAs more highly expressed in the LW strain. Red indicates miRNAs more highly expressed in the SW strain. (**B**,**C**) Venn diagrams showing the overlap of miRNAs more highly expressed in the LW (**B**) and SW (**C**) strains at different instar stages (Differential expression analysis by DESeq2, adjusted *p*-value < 0.05, fold change > 2).

To investigate the effect of miRNAs on the regulation of wing morph plasticity, we first calculated the reads counts for each miRNA and then used DESeq2 [38] to compare miRNA expression in the LW and SW BPH strains at the same stage (Supplementary Table S2). A total of 122 miRNAs were identified as being differentially expressed between the two strains during nymph development (Figure 2A and Supplementary Table S3). Among them, 94 miRNAs were differentially expressed in the 1st instar, including 47 miRNAs more highly expressed in LW strain and 47 miRNAs more highly expressed in the SW strain. In the 4th instar nymph, 27 miRNAs were more highly expressed in the LW strain while 29 miRNAs were more highly expressed in the SW strain. In the 5th instar, 41 miRNAs were more highly expressed in the LW strain, while only 25 miRNAs were more highly expressed in the SW strain. Moreover, in the 3rd instar, only two miRNAs were more highly expressed in the LW strain and three miRNAs were more highly expressed in the SW strain. No miRNAs were differentially expressed in the 2nd instar (Figure 2B). 

In particular, *Nlu-nov-5-5p* was significantly more highly expressed in all four instar stages of LW BPHs, while *Nlu-nov-26-5p* showed a more complicated expression pattern, with a high level of expression in the 1st and 4th instar stages of the SW strain and high expression in the 3rd and 5th instar stages of the LW strain (Supplementary Figure S1). Interestingly, 13 miRNAs were significantly more highly expressed in the 1st and 4th instar stages but significantly more lowly expressed in 5th instar stage of the LW strain compared with the SW strain. However, another 19 miRNAs showed a similar expression pattern (i.e., higher expression in the 1st and 4th instar but lower expression in the 5th instar) when comparing the SW strain with the LW strain. The presence of these differentially expressed miRNAs implies that miRNAs are probably associated with the regulation of wing plasticity in BPH.

### 2.3. Functional Analysis of Differentially Expressed miRNAs

**Figure 3:**
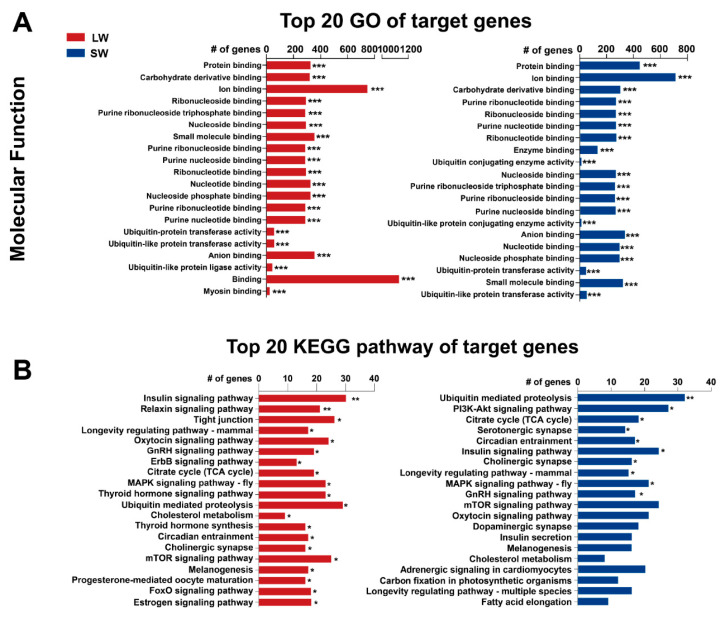
Gene Ontology (GO) and Kyoto Encyclopedia of Genes and Genomes (KEGG) enrichment analyses of targets of differentially expressed miRNAs. (**A**) GO molecular function terms enriched in differentially expressed miRNA-targeted genes in LW and SW strains. (**B**) KEGG pathways enriched in differentially expressed miRNA-targeted genes in LW and SW strains. * *p* < 0.05, ** *p* < 0.01, *** *p* < 0.001.

To further investigate the roles of these differentially expressed miRNAs, we used four algorithms, miRanda [39], TargetScan [40], RNAhybrid [41,42] and PITA [43], to predict the miRNA targets. In total, 1907 and 1724 genes were predicted as targets of the miRNAs more highly expressed in the LW and SW strains, respectively (Supplementary Tables S4 and S5). In both the LW and SW BPH strains, the predicted target genes were mainly annotated to GO terms related to binding activity, such as protein binding, carbohydrate derivative binding, ion binding, ubiquitin protein ligase binding and nucleoside- and purine-related binding activities (Figure 3A and Supplementary Tables S6 and S7). In particular, miRNAs more highly expressed in LW strain BPH also participated in the regulation of protein activities, including nucleoside-triphosphatase activity (GO:0017111, adjusted *p*-value = 2.66 × 10^−5^), pyrophosphatase activity (GO:0016462, adjusted *p*-value = 4.13 × 10^−5^), primary active transmembrane transporter activity (GO:0015399, adjusted *p*-value = 1.32 × 10^−4^) and ATPase activity (GO:0016887, adjusted *p*-value = 1.44 × 10^−4^) (Supplementary Tables S6 and S7). 

KEGG pathway enrichment analysis showed that target genes of all the miRNAs more highly expressed in the LW strain were significantly enriched in 32 pathways, including the insulin signaling pathway (KO04910, adjusted *p*-value = 0.0017), MAPK signaling pathway (KO04013, adjusted *p*-value = 0.017), mTOR signaling pathway (KO04150, adjusted *p*-value = 0.028), FoxO signaling pathway (KO04068, adjusted *p*-value = 0.030), thyroid hormone signaling pathway (KO04919, adjusted *p*-value = 0.018) and thyroid hormone synthesis (KO04918, adjusted *p*-value = 0.024) (Figure 3B and Supplementary Table S8). Only 10 pathways were significantly enriched for the targets of all the miRNAs more highly expressed in the SW strain and all were among the 32 enriched pathways in the LW strain (Figure 3B and Supplementary Table S9). Notably, targets of differentially expressed miRNAs in both strains included those involved in the endocrine system (insulin signaling pathway and gonadotropin-releasing hormone (GnRH) signaling) and signal transduction (PI3K-Akt signaling pathway and MAPK signaling pathway), implying their vital roles in the regulation of wing morph plasticity. Moreover, some specific pathways only enriched in the LW strain are speculated to be responsible for the development of winged morphs; for example, the Hippo signaling pathway (KO04390, adjusted *p*-value = 0.050) was found to participate in morphogen control of wing growth in *Drosophila* [44,45,46]. Interestingly, the insulin signal transduction pathway was only significantly enriched in the 5th instar nymph of the LW strain (KO04910, adjusted *p*-value = 5.89 × 10^−3^); however, insulin signaling (KO04910, adjusted *p*-value = 9.71 × 10^−3^ in the 1st instar and 1.63 × 10^−2^ in the 4th instar) and related transduction pathways such as the PI3K-Akt signaling pathway (KO04151, adjusted *p*-value = 9.71 × 10^−3^ in the 4th instar) and MAPK signaling pathway (KO04013, adjusted *p*-value = 3.03 × 10^−2^ in the 1st instar and 1.63 × 10^−2^ in the 4th instar) were enriched in specific nymph stages of the SW strain (Supplementary Tables S10 and S11). Moreover, thyroid hormone synthesis (KO04918, adjusted *p*-value = 1.33 × 10^−2^) was only significantly enriched in the 5th instar of the LW strain and was also identified as significantly enriched in the targets of highly expressed miRNAs from all LW stages. This indicates that miRNAs participate in the modulation of wing type plasticity via insulin signal transduction and that there is complicated endocrine regulation at different stages of BPH development.

### 2.4. Validation of miRNAs and Target Gene

**Figure 4:**
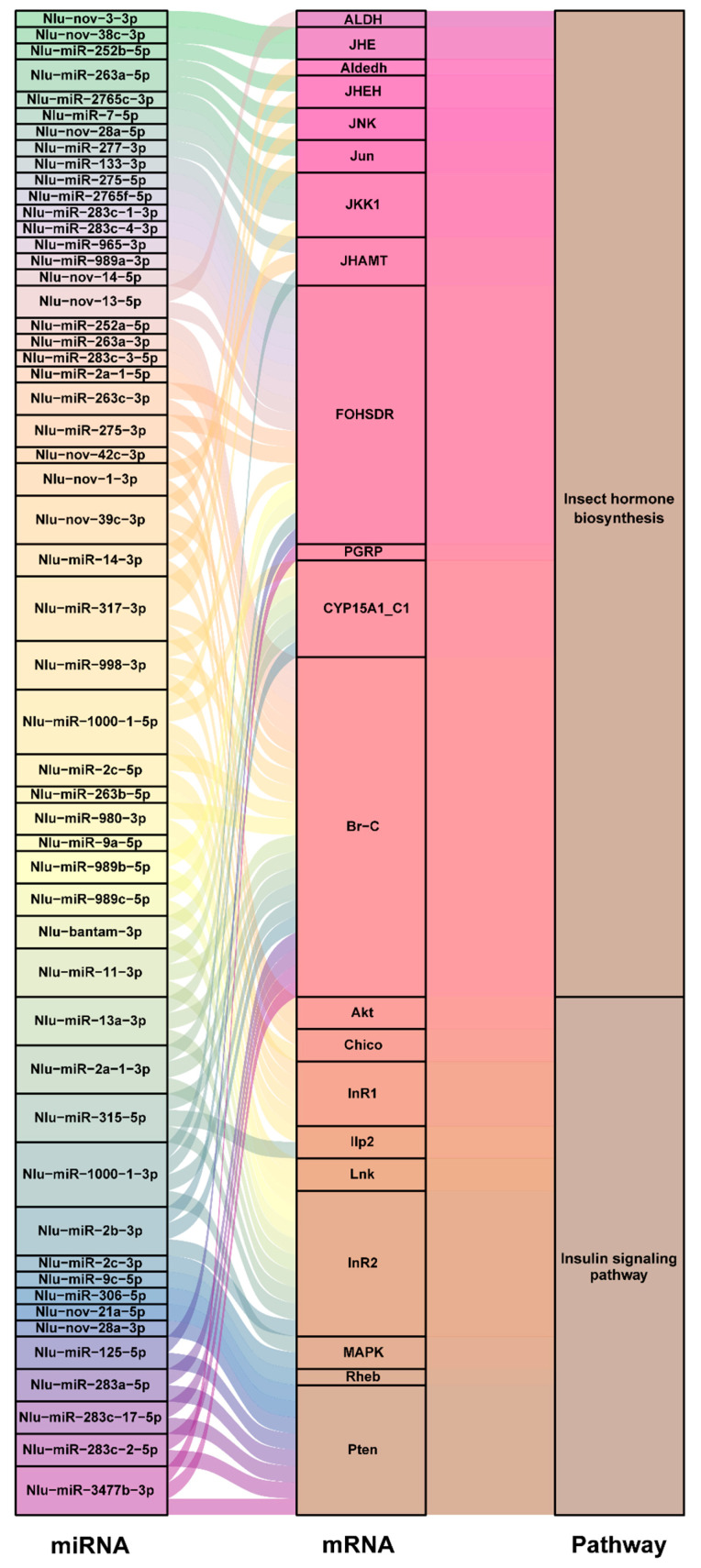
Sankey diagrams of miRNAs, target genes and enriched pathways predicted by more than two algorithms (miRanda, TargetScan, RNAhybrid and PITA). *ALDH*, Aldehyde dehydrogenase dimeric NADP preferring; *JHE*, Juvenile hormone esterase; *Aldedh*, Aldehyde dehydrogenase cytosolic 2-like; *JHEH*, Juvenile hormone epoxide hydrolase; *JNK*, Mitogen-activated protein kinase 8/9/10 (c-Jun N-terminal kinase); *JUN*, Transcription factor AP-1; *JKK1*, Dual specificity mitogen-activated protein kinase kinase 7-like; *JHAMT*, Juvenile hormone-III synthase; *FOHSDR*, Farnesol dehydrogenase-like; *PGRP*, Peptidoglycan recognition protein; *CYP15A1_C1*, Methyl farnesoate epoxidase-like; *Br-C*, Broad-complex core protein; *Akt*, Serine/threonine-specific protein kinase; *Chico*, Insulin receptor substrate; *InR1*, Insulin receptor 1; *Ilp2*, Insulin-like peptide; *Lnk*, SH2B adapter protein 1-like protein; *InR2*, Insulin receptor 2; *MAPK,* Mitogen-activated protein kinase; *Rheb*, Ras homolog enriched in brain; *Pten*, *Phosphatidylinositol-3,4,5-trisphosphate 3-phosphatase.*

**Figure 5:**
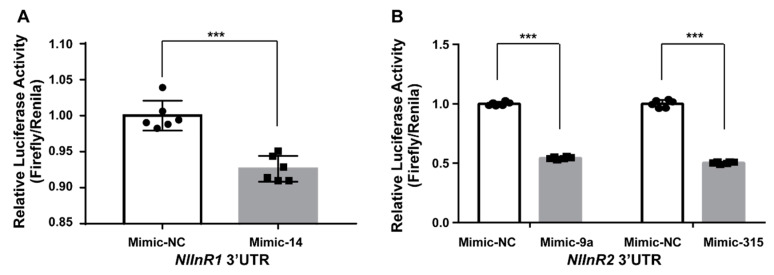
Dual luciferase reporter assays confirmed the interactions between miRNAs and *NlInR* transcripts in vitro. (**A**) The interaction of *Nlu-miR-14-3p* with *NlInR1*. (**B**) The interaction of *Nlu-miR-9a-5p* and *Nlu-miR-315-5p* with *NlInR2*. In the presence of mimic, the relative luciferase activities were significantly decreased compared with the negative control (NC) group. The black circulars and black squares are presented as 6 replicates. Data are means ± SEM (*n* = 6). *** *p* < 0.001.

To further validate the interaction of miRNAs and target genes involved in insulin signal transduction pathways, we first searched for the target genes of all the differentially expressed miRNAs in this pathway. Since insect hormone-related genes are involved in insect development and particularly in wing polymorphism in insects [47,48,49,50,51], the complete sequences of these genes were also included in the target input file. We found that 26 miRNAs shared more than one target gene and that 22 miRNAs had target genes in both the insulin and insect hormone pathways. The *broad-complex core protein* (*Br-C*) gene, which is an upstream factor in the insulin signaling pathway [32], was targeted by as many as 21 miRNAs and the *insulin receptor* (*NlInR*) genes were targeted by 13 miRNAs (Figure 4 and Supplementary Table S12). Since insulin receptors control wing plasticity in BPH [11], three miRNAs, *Nlu-miR-14-3p*, *Nlu-miR-9a-5p* and *Nlu-miR-315-5p*, which are predicted to target the insulin receptors, were selected to confirm the interaction between miRNAs and target genes in dual luciferase reporter assays in vitro. In the presence of miRNA mimic, luciferase activities decreased significantly compared with those of the negative control (Figure 5). These results indicate that these miRNAs might modulate the wing morph of BPHs via the insulin signaling pathway and insect hormone regulation.

## 3. Discussion

Wing polymorphism is a successful strategy allowing BPHs to adapt different environmental factors [10]. Multiple factors play pivotal roles in controlling wing polyphenism, particularly those functioning in the IIS signaling pathway and endocrine biosynthesis in insects [10]. However, whether miRNAs regulate the phenotypic plasticity of the wing via these pathways is seldom investigated. Here, we focused on identifying miRNAs regulating wing determination at different developmental stages of BPHs. By sequencing small RNA libraries, we identified 254 miRNAs, including 158 conserved and 96 novel miRNAs, which is similar to the total number of miRNAs reported in a previous study of BPH [36]. Moreover, we identified 122 miRNAs that were differentially expressed (>2-fold change) between the LW and SW strains in at least one of four nymph instar stages (1st, 3rd, 4th and 5th instars). This suggests that these miRNAs are associated with the regulation of wing polyphenism in BPH. Interestingly, differentially expressed miRNAs in 1st instar shared similar expression pattern to those in 4th instar with the heatmap (Figure 2A). And we also found 24 miRNAs in both two instars significantly highly expressed in LW strain, while 26 miRNAs showed similar pattern in SW strain. Additionally, 22 of 23 other highly expressed miRNAs in 1st instar of LW strain, also highly expressed in 4th instar with the low fold change or no significant difference (*p*-value > 0.05) (Supplementary Table S2). In SW strain, 20 of 21 other highly expressed miRNAs in 1st instar were in the same situation (Supplementary Table S2). This result indicates that wing polymorphism in BPH may be regulated via similar miRNAs in these two instars when compared with that in 2nd and 3rd instars. However, the reason for this similar expression pattern is still unclear and it needs further investigation to elucidate the roles of these miRNAs.

Differentially expressed miRNAs likely regulate wing polymorphism via multiple pathways at different instar stages in these two strains. In the 1st and 4th instar nymphs, targets of highly expressed SW miRNAs were significantly enriched in multiple pathways, such as adrenergic signaling in cardiomyocytes (KO04261), fatty acid metabolism (KO01212), dopaminergic synapse (KO04728) and the pyruvate metabolism pathway (KO00620). Polyunsaturated fatty acids are reported to play crucial roles in insect reproduction [52,53]. And in a previous study, a reduction in synaptic vesicular release was associated with *Drosophila* flight deficits [54]. The lower expression of genes in these pathways may lead to the high reproduction and weak flight ability in SW strain. In contrast, in the 3rd instar nymphs, the targets of miRNAs more highly expressed in the LW strain were significantly enriched in the sphingolipid (KO04071) and HIF-1 (KO04066) signaling pathways. Sphingolipid signaling is involved in insect development via wingless signaling and the *sphingosine-1-phosphate lyase* homolog *Sply* regulates the pattern of the dorsal longitudinal flight muscles of the *Drosophila* adult thorax [55,56]. Nymphal miRNAs might regulate the sphingolipid signaling pathway to strengthen the dorsal longitudinal flight muscles allowing for long-distance migration of the LW BPH strain. In the 5th instar nymphs, targets of miRNAs more highly expressed in the LW strain were enriched in multiple pathways, such as the regulation of lipolysis in adipocytes (KO04923), thyroid hormone synthesis (KO04918) and the glucagon signaling pathway (KO04922) and the targets of miRNAs more highly expressed in the SW strain were enriched in the longevity regulating (KO04211), mTOR signaling (KO04150) and glycosphingolipid biosynthesis (KO00604) pathways. In addition, some pathways were differentially regulated between the LW and SW strains at different instar stages. For instance, the MAPK signaling (KO04013) and insulin signaling (KO04910) pathways were significantly enriched in differentially expressed miRNA target genes in 1st and 4th instar SW nymphs and in 5th instar LW nymphs. The results indicated that the expression of miRNAs varies between instars and that miRNAs regulate wing dimorphism of BPH in a specific and complicated manner by targeting genes at different developmental stages in the SW and LW strains.

Previously, the tight junction pathway (KO04530) was found to be enriched in differentially expressed genes across the egg, nymph and adult stages between LW and SW strain BPHs [19]. In our study, the tight junction pathway was also significantly enriched in the targets of miRNAs highly expressed in the LW strain (adjusted *p*-value = 1.18 × 10^−2^) (Supplementary Table S8). Moreover, targets of miRNAs more highly expressed in the LW strain were also enriched in vascular smooth muscle contraction (KO04270, adjusted *p*-value = 3.80 × 10^−2^) and the oxytocin signaling pathway (KO04921, adjusted *p*-value = 1.62 × 10^−2^) (Supplementary Table S8). Zhang et al. [19] also found that differentially expressed genes between LW and SW strain at the 3rd instar nymph stage were significantly enriched in vascular smooth muscle contraction and the oxytocin signaling pathway. These results imply that the regulation of miRNA expression might be involved in the determination of wing length.

Among the differentially expressed miRNAs, 53 were predicted to target numerous genes in the insulin signaling pathway or insect hormone biosynthesis-related pathways and 22 of these miRNAs were predicted to target genes in both pathways (Supplementary Table S12). Therefore, we speculate that miRNAs mediate crosstalk between the IIS pathway and insect hormones to modulate the development of wing morphs. Although *Nlu-miR-34* was not included in the list of differentially expressed miRNAs because it only showed a 1.7-fold change in expression between the LW and SW BPH strains at the whole-body level, the expression in wing buds may be higher in the SW strain. This implies that tissue-specific expression of miRNAs might play a vital role in regulating the JH, 20E and IIS pathways at key stages where wing morph is determined—the 2nd to 4th instar nymph stages [32]. A dual luciferase reporter assay confirmed the interaction of *NlInR* with three miRNAs, *Nlu-miR-14-3p*, *Nlu-miR-9a-5p* and *Nlu-miR-315-5p*, in vitro; however, further evidence from in vivo overexpression or knock-down experiments are needed to clarify the functions of these miRNAs in the regulation of wing plasticity.

In previous study, Yang et al. [57] found that *miR-1000* regulates morphogenesis of the wing and compound eyes in the American cockroach. The fact that we identified *Ultrabithorax* (*NlUbx*, MK855103.1) as a potential target of the differentially expressed miRNA *Nlu-miR-1000-1-3p*, which regulates wing dimorphism by regulating host nutrition status [15], provides evidence that *Nlu-miR-1000* plays an important role in wing polyphenism. In addition, the targets of differentially expressed miRNAs in the LW strain were also significantly enriched in the Hippo signaling pathway, which is regarded as a conserved regulator of organ size in *Drosophila* [46,58]. *Nlu-miR-263a-5p* is predicted to target *protein expanded*, which is a component of the Hippo signaling pathway (Supplementary Tables S4 and S5). Protein expanded is a mediator between *fat* and the Hippo signaling pathway and *fat* participates in morphogen control of wing growth of *Drosophila* [44,45,46]. This indicates that *Nlu-miR-263a-5p* may regulate wing development polymorphism by targeting *protein expanded*.

## 4. Materials and Methods 

### 4.1. Insect Rearing

The BPHs were originally collected in rice fields of Wuhan, Hubei Province, China and maintained in a climate room at 26 ± 1 °C under a 16L:8D light regime with a relative humidity of 75 ± 5%. New seedlings of the susceptible rice variety Taichuang Native 1 (TN1) were used for feeding. To obtain the LW and SW strains, in each generation LW and SW individuals from the LW and SW populations, respectively, were selected at the adult stage as described in a previous study [59] and selection was carried out for more than 50 generations. The rates of LW and SW BPHs in the corresponding strains used for experiments were at least 80%.

### 4.2. Small RNA Library Preparation and Sequencing 

The whole body of BPH nymphs from the 1st to 5th instars (day 1 of each stage) were collected for small RNA sequencing with two biological replicates. Total RNA was extracted with TRIzol. The RNA degradation and contamination were monitored on 1% agarose gels. The purity, concentration and integrity of RNA were tested by NanoPhotometer spectrophotometer (IMPLEN, Los Angeles, USA), Qubit 2.0 fluorometer (Life Technologies, Carlsbad, USA) and Agilent Bioanalyzer 2100 system (Agilent Technologies, Palo Alto, USA), respectively. 3 µg RNA per sample was used as input to generate the sequencing libraries using the NEBNext Multiplex Small RNA Library Prep Kit for Illumina (NEB, Ipswich, USA) following the manufacturer’s protocol. After ligating the 3′ SR adaptor and 5′ SR adaptor to the ends of the small RNAs, M-MuLV Reverse Transcriptase (RNase H^−^) was used to synthesize the first strand cDNA. Then, PCR amplification was performed and the products were purified by electrophoresis on an 8% polyacrylamide gel at 100 V for 80 min. Fragments of 140–160 bp were recovered and the library quality was assessed using DNA High Sensitivity Chips (Agilent Technologies, Palo Alto, USA) on the Agilent Bioanalyzer 2100 system. The library preparations were sequenced at Novogene (Beijing, China) on the Illumina HiSeq 2500/2000 platform.

### 4.3. miRNA Prediction

To identify miRNAs, adaptors and low-quality reads were first removed from the raw data for each small RNA library, then the clean data for all the libraries were combined together and analyzed using two algorithms, miRDeep2 [33,34] and MapMi [35]. For miRDeep2, mapper.pl was used to map the reads to the reference genome (parameters: -c -j -l 18 -m -p BPH -s -t -v; discarding reads shorter than 18 nt). Conserved and novel miRNAs were identified by miRDeep2.pl with the miRBase mature sequence [60] (the minimum cut-off score was set by 5 and significant Randfold *p*-value is set as yes). MapMi was performed with the cut-off score more than 25 and minimum free energy (MFE) less than −18 kcal/mol. The high quality BPH genome sequence (accession number GCA_014356525.1) [61] was used as a reference. miRNAs predicted by both algorithms were retained for further analysis after removing redundant sequences. After comparing to the miRNAs in miRBase, unmatched miRNA candidates were filtered with the criterion described in miRBase [60] and then were identified as novel miRNAs.

### 4.4. Differential Expression Analysis 

To identify miRNAs differentially expressed in each library, miRNA frequencies were calculated using a Perl script after removing the miRNAs with a low number of reads (total count for all samples < 50). In the same instar of each strain, the miRNA counts of two libraries were compared by R package ggplot2 (Supplementary Figure S2) and it indicated that the expression of miRNAs was highly reproducible between biological replicates. Then the pairwise comparison of the filtered data was analyzed using the R package DESeq2 with a standard workflow [38]. Only those miRNAs with greater than a 2-fold change (FC > 2) in expression and a significant false discovery rate (adjusted *p*-value < 0.05) were determined to be significantly differentially expressed between the corresponding nymph stages of the two strains.

### 4.5. Target Gene Prediction and GO and KEGG Enrichment Analysis 

To predict the target genes of differentially expressed miRNAs, 3′ untranslated region (UTR) sequences of BPH were downloaded from InsectBase [62]. Complete sequences of genes in BPH wing polymorphism-related pathways such as the insect hormone biosynthesis pathway (KO00981) and insulin signaling pathway (KO04910) were identified and downloaded from the National Center for Biotechnology Information (NCBI, Bethesda, USA) (Supplementary Table S13). Next, four algorithms, miRanda [39], TargetScan [40], RNAhybrid [41,42] and PITA [43] were used for miRNA prediction. The energy minimum was set at <−20 kcal/mol for prediction by miRanda, while targets filtered with *p*-value < 0.05 were retained by RNAhybrid. TargetScan and PITA were run with the default parameters. The miRNA-target pairs predicted by all four algorithms were kept for further analysis. The target genes of differentially expressed miRNAs were annotated to GO terms using Blast2GO software [63] and mapped to KEGG pathways using the BlastKOALA web server (https://www.kegg.jp/blastkoala/). NCBI non-redundant protein sequences database was used in Blast2GO to annotate GO number of miRNA targets with blastx configuration. In BlastKOALA web server, miRNA targets were blasted with animal group datasets. With the results of GO number and KEGG number of targets, the enrichment analysis was analyzed and visualized on the cloud platform of OmicShare (http://www.omicshare.com/tools). The background of enrichment is the whole annotation genes in BPH. For each strain, we combined the target genes of highly expressed miRNAs among four instars and performed strain specific enrichment of GO terms and KEGG pathways (Supplementary Tables S6–S9). For highly expressed miRNAs in each instar stage, the KEGG pathways enrichment of their target genes was also analyzed (Supplementary Tables S10 and S11). All results were filtered with the adjusted *p*-value ≤ 0.05.

### 4.6. In Vitro Luciferase Assays 

The 3′UTR sequences of the target genes *NlInR1* and *NlInR2* were cloned downstream of the firefly luciferase gene in the pMIR-REPORT vector (Obio, Shanghai, China). The *Renilla* luciferase gene in the pRL-CMV vector (Promega, Madison, USA) was used as a control luciferase reporter. HEK293T cells (Obio, Shanghai, China) were cultured for 24 h in 96-well culture plates with DMEM (Gibco, Carlsbad, USA) and 10% FBS (Hyclone, Logan, USA) at 37 °C and 5% CO_2_. miRNA mimics (RiboBio, Guangzhou, China) were added to Lipofectamine 2000 (Invitrogen, Carlsbad, USA) at a concentration of 100 nM for transfection. A random sequence miRNA mimic, UUUGUACUACACAAAAGUACUG, was used as the negative control. Cells were transfected with a transfection reagent mixture of pMIR-REPORT: pRL-CMV: Lipofectamine 2000 at a ratio of 0.2 μg: 0.01 μg: 0.25 μL following the manufacturer’s instructions. At 48 h post transfection, the activities of the two luciferase enzymes were measured on an Infinite M1000 (Tecan, Männedorf, Switzerland) with six replicates. The ratio of the mean firefly luciferase activity to mean *Renilla* luciferase activity (Firefly/*Renilla*) was calculated to obtain the relative luciferase activity and ratios were compared using the two-tailed Student’s *t*-test.

## 5. Conclusions

The significance of miRNAs function in wing polymorphism will continue to grow as more genes and pathways identified to regulate the development of wing morphs. In this work, we constructed small RNA libraries of all nymph instars of two BPH strains with different wing types and revealed a complex association of miRNAs and their regulatory network ensuring the development of distinct wing morphs between the LW and SW strains of BPH. miRNAs differentially expressed at specific stages regulate wing polyphenism mainly via two mechanisms, the regulation of gene expression levels and hormone titers. For gene expression level, miRNAs regulate the wing morphs via the insulin signaling pathway and some other pathways related to the flight ability (like sphingolipid signaling pathway), the reproduction ability (like dopaminergic synapse pathway). For hormone titers, miRNAs could function in the biosynthesis of JH, 20E and other hormones in insects. The expression of miRNAs varies between instars but 1st and 4th instars shared similar expression pattern. We will analyze the relationship of these two instars in future. And regulation of miRNAs in wing dimorphism is also complicated and specific at different developmental stages in two strains. These findings provide a candidate gene set, which will be helpful for mining the key factors involved in the determination of wing plasticity.

## Figures and Tables

**Table 1:** Reads obtained from small RNA libraries sequenced in *Nilaparvata lugens*.

No	Sample	Raw Reads	High Quality	Clean Reads	Q20	GC Content	5′adapter Contamines	3′adapter_Null or Insert_Null	with PloyA/T/G/C
1	LW-1st-1	13,625,214	13,614,741	13,330,526	98.25%	48.45%	3519	217,585	62,872
2	LW-1st-2	14,390,780	14,380,460	14,079,327	98.33%	47.56%	2826	234,820	63,230
3	LW-2nd-1	13,447,515	13,437,862	13,204,811	98.25%	47.64%	2869	162,612	67,300
4	LW-2nd-2	12,552,925	12,544,080	12,284,953	98.22%	47.33%	2611	189,043	67,244
5	LW-3rd-1	14,008,106	13,997,085	13,723,894	98.40%	47.89%	3182	194,273	75,452
6	LW-3rd-2	13,190,404	13,178,303	12,975,678	98.22%	48.60%	3609	149,545	49,216
7	LW-4th-1	18,344,628	18,328,565	17,708,977	98.13%	47.87%	5209	540,722	73,320
8	LW-4th-2	18,656,643	18,637,940	17,960,139	98.11%	48.57%	4572	603,621	69,249
9	LW-5th-1	17,176,623	17,165,563	16,607,021	98.32%	47.57%	5806	474,728	77,696
10	LW-5th-2	16,751,585	16,739,158	16,254,637	98.12%	47.58%	5980	419,723	58,517
11	SW-1st-1	17,257,604	17,247,057	16,612,101	98.16%	47.29%	8058	505,257	121,319
12	SW-1st-2	13,757,659	13,748,950	13,172,776	98.21%	46.95%	5890	466,836	103,188
13	SW-2nd-1	15,251,104	15,240,012	14,434,758	98.28%	47.38%	3383	683,287	118,280
14	SW-2nd-2	19,765,649	19,750,938	19,257,968	98.33%	47.36%	4397	344,244	143,995
15	SW-3rd-1	18,780,777	18,766,573	17,960,404	98.41%	48.60%	5290	696,703	103,821
16	SW-3rd-2	16,793,873	16,779,788	16,026,154	98.26%	48.51%	4287	666,676	82,349
17	SW-4th-1	13,125,238	13,116,371	12,854,273	98.26%	48.00%	7247	142,684	111,947
18	SW-4th-2	13,552,267	13,541,969	13,173,223	98.12%	47.65%	4728	229,093	134,671
19	SW-5th-1	19,598,809	19,582,736	18,883,495	98.21%	47.47%	4252	567,450	127,157
20	SW-5th-2	19,297,940	19,282,397	18,584,543	98.29%	47.92%	4342	573,153	120,010

LW: long-wing strain, SW: short-wing strain, High quality: raw reads without low quality reads, Q20: the proportion of bases with phred value > 20, 5′adapter contamines: reads with 5′adapter, 3′adapter_null or insert_null: reads without 3′adapter or insertion, with ployA/T/G/C: reads with ployA/T/G/C.

## Supplementary Materials

The following are available online at https://www.mdpi.com/1422-0067/21/24/9754/s1, Supplementary materials are uploaded.

## Abbreviations

LW	Long-wing
SW	Short-wing
GO	Gene Ontology
KEGG	Kyoto Encyclopedia of Genes and Genomes
BPH	Brown planthopper
NCBI	National Center for Biotechnology Information
Akt	Serine/threonine-specific protein kinase
Aldedh	Aldehyde dehydrogenase cytosolic 2-like
ALDH	Aldehyde dehydrogenase dimeric NADP preferring
Br-C	Broad-complex core protein
Chico	Insulin receptor substrate
CYP15A1_C1	Methyl farnesoate epoxidase-like
FOHSDR	Farnesol dehydrogenase-like
GnRH	Gonadotropin-releasing hormone
IIS	Insulin/insulin-like growth factor signaling
Ilp2	Insulin-like peptide
InR1	Insulin receptor 1
InR2	Insulin receptor 2
JH	Juvenile hormone
JHAMT	Juvenile hormone-III synthase
JHE	Juvenile hormone esterase
JHEH	Juvenile hormone epoxide hydrolase
JKK1	Dual specificity mitogen-activated protein kinase kinase 7-like
JNK	Mitogen-activated protein kinase 8/9/10 (c-Jun N-terminal kinase)
JUN	Transcription factor AP-1
Lnk	SH2B adapter protein 1-like protein
MAPK	Mitogen-activated protein kinase
PGRP	Peptidoglycan recognition protein
Pten	Phosphatidylinositol-3,4,5-trisphosphate 3-phosphatase
Rheb	Ras homolog enriched in brain
