# Supplementary figures and images for: Identification and Analysis of MicroRNAs Associated with Wing Polyphenism in the Brown Planthopper, Nilaparvata lugens
Source: Int J Mol Sci. 2020 Dec 21;21(24):9754. doi: 10.3390/ijms21249754 (PMC7767257; doi:10.3390/ijms21249754)

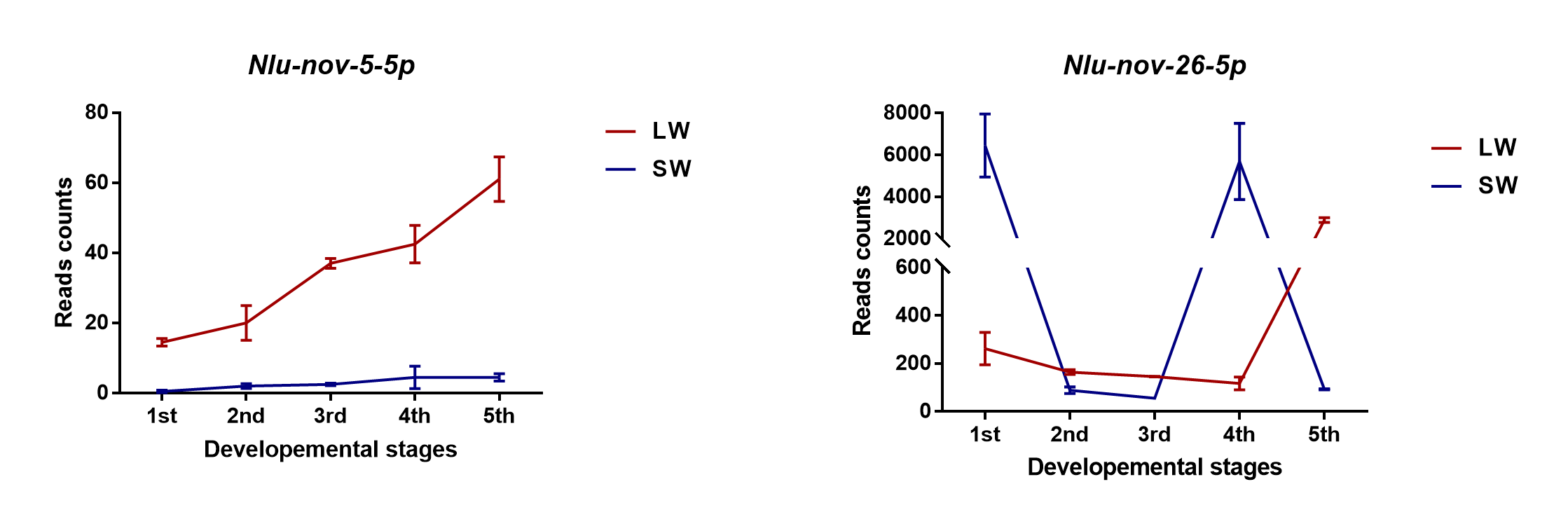

Supplement: Supplementary file 1 [file ijms-21-09754-s001.zip › ijms-supplementary-20201214/Supplement_Figure_S1_Expression of specific miRNAs.tif]

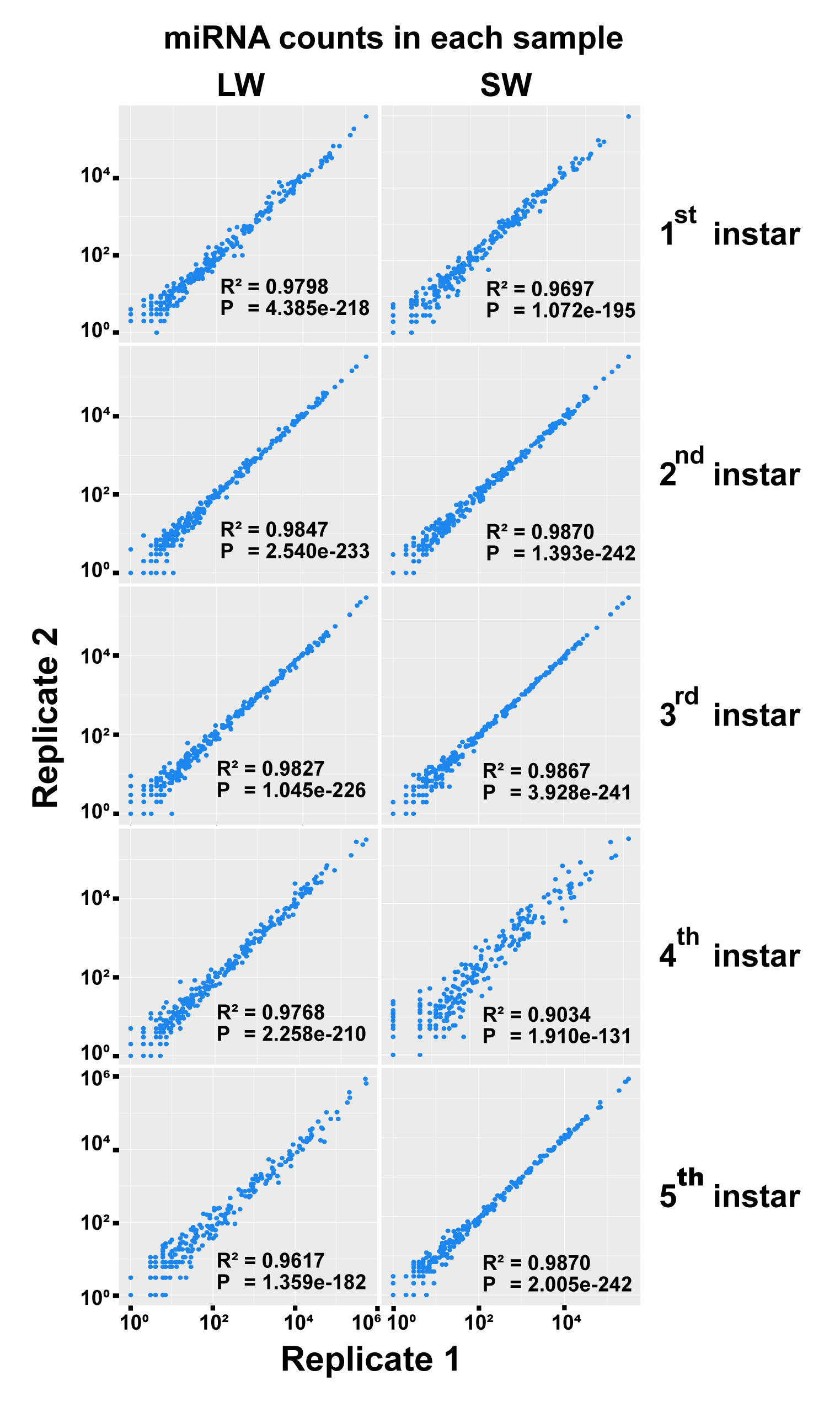

Supplement: Supplementary file 1 [file ijms-21-09754-s001.zip › ijms-supplementary-20201214/Supplement_Figure_S2_miRNA_counts_in_each_sample.tif]
